# A stable room-temperature sodium–sulfur battery

**DOI:** 10.1038/ncomms11722

**Published:** 2016-06-09

**Authors:** Shuya Wei, Shaomao Xu, Akanksha Agrawral, Snehashis Choudhury, Yingying Lu, Zhengyuan Tu, Lin Ma, Lynden A. Archer

**Affiliations:** 1 School of Chemical and Biomolecular Engineering, Cornell University, Ithaca, New York 14853, USA; 2 College of Chemical and Biological Engineering, Zhejiang University, Hangzhou 310027, China; 3 Department of Material Science and Engineering, Cornell University, Ithaca, New York 14853, USA

## Abstract

High-energy rechargeable batteries based on earth-abundant materials are important for mobile and stationary storage technologies. Rechargeable sodium–sulfur batteries able to operate stably at room temperature are among the most sought-after platforms because such cells take advantage of a two-electron-redox process to achieve high storage capacity from inexpensive electrode materials. Here we report a room-temperature sodium–sulfur battery that uses a microporous carbon–sulfur composite cathode, and a liquid carbonate electrolyte containing the ionic liquid 1-methyl-3-propylimidazolium-chlorate tethered to SiO_2_ nanoparticles. We show that these cells can cycle stably at a rate of 0.5 C (1 C=1675, mAh g^−1^) with 600 mAh g^−1^ reversible capacity and nearly 100% Coulombic efficiency. By means of spectroscopic and electrochemical analysis, we find that the particles form a sodium-ion conductive film on the anode, which stabilizes deposition of sodium. We also find that sulfur remains interred in the carbon pores and undergo solid-state electrochemical reactions with sodium ions.

## Abstract (web summary)

Rechargeable sodium-sulfur batteries able to operate stably at room temperature are sought-after platforms as they can achieve high storage capacity from inexpensive electrode materials. Here, the authors use rationally selected cathode and electrolyte materials to design a room temperature Na-S battery.

## Graphical abstract

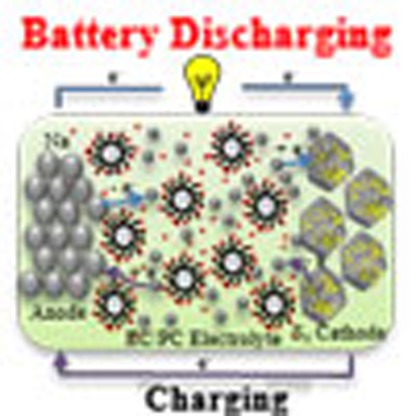

The importance of rechargeable lithium batteries in portable electronics and their potential for electrifying transportation have been well described in several reviews^1^,^2^,^3^,^4^. Various recent efforts have focused on the lithium–sulfur (Li–S) chemistry due to the high theoretical-specific energy (2,500 W h kg^−1^), high natural abundance and environmental benignity of the sulfur cathode, with great progress being achieved during the past decade^5^,^6^,^7^,^8^,^9^,^10^,^11^. Although many technical challenges remain, the cost and feasibility of batteries that use metallic lithium as the anode and sulfur as the cathode appear good for applications in transportation, but less so for grid-related applications, where scale and cost are as important as performance^12^,^13^. Sodium, the second lightest and smallest alkali metal is a low-cost alternative to lithium as anode and is available in regions all over the world, it is therefore unsurprising that interest in Na-based batteries predate those in Li-based ones^14^,^15^.

High-temperature sodium–sulfur (Na–S) batteries operated at >300 °C with molten electrodes and a solid β-alumina electrolyte have been commercialized for stationary-energy-storage systems, confirming that this cell chemistry can meet the scale and cost requirements for feasibility in grid-scale applications^16^,^17^. A stable room-temperature analogue of the rechargeable Na–S battery with a higher theoretical-specific energy of 1,274 W h kg^−1^ (refs 18, 19) has to date proven remarkably elusive, despite its superficial analogies to room-temperature Li–S batteries under active study. The large difference in size between Na atom and Na^+^ ion defines one aspect of the challenge, as it is thought to make sodium more prone than lithium to form unstable electrodeposits and dendrites^15^. Sodium is also more reactive with aprotic liquid electrolyte solvents and forms a less-stable protective solid electrolyte interface (SEI) in aprotic liquids^18^,^20^, which leads to lower electrochemical conversion efficiency. Na^+^ ions are larger and less reducing than Li^+^ ions^15^, which implies that transport and kinetics of electrochemical processes in the cathode are more sluggish. Finally, Na reduction products with sulfur are more soluble than the analogous ones for lithium^14^. Taken together, these traits mean that a successful Na–S cell must overcome multiple new challenges, in addition to the already well-known ones facing Li–S batteries: the insulating nature of sulfur and its solid-state discharge product; the solubility of intermediate lithium polysulfides (LiPS) species and their associated shuttling between the electrodes, which lowers the Coulombic efficiency of the cell; and volume expansion of the cathode on cell discharge^6^,^7^,^21^,^22^. It is significant that some of these problems remain even when a solid-state electrolyte is employed in high-temperature Na–S cells in which the Na metal anode is a liquid.

To construct a room-temperature rechargeable Na–S battery, a conductive cathode substrate able to overcome the electronically insulating nature of both the fully charged and discharged products (S and Na_2_S) is required for high active material utilization. To maintain stable cell performance, the substrate must also be able to prevent loss of the intermediate sodium polysulfides (NaPS) species^23^ to the electrolyte. Sulfur infused into microporous carbon materials with small pore sizes (*d*_p_<1.8 nm) and high surface areas (*S*_A_≥843 m^2^ g^−1^) have been reported previously^19^,^24^,^25^,^26^,^27^,^28^. When employed as cathodes in Li–S cells, the materials have been reported to display only one of the two discharge plateaus observed in traditional Li–S batteries, which has been argued to lend support to the hypothesis that in microporous carbons sulfur undergoes a solid-state electrochemical reaction with Li^+^ to form solid sulfide product species in the cathode—that is, without forming soluble LiPS^25^,^28^. Xin *et al*.^19^,^26^ have presented an alternative argument that supports formation of smaller sulfur (S_2–4_) species in microporous carbon substrates that on reduction with Li^+^ cannot form soluble high-order LiPS. Although this argument is a reasonable interpretation of the electrochemistry data, support from thermodynamic analysis of the electrode has been lacking so far. On the anode side, fundamentally based strategies for preventing dendrite formation in lithium metal batteries should necessarily be applicable for the sodium anode. Among the most fundamental of these approaches, are the efforts reported by Schaefer *et al*.^29^,^30^ and Lu *et al*.^31^,^32^,^33^,^34^ to reduce the magnitude of destabilizing electric fields near the anode by tethering anions to slow-moving or immobile supports^35^,^36^. Other successful methods, such as introduction of LiF^37^ or fluoro-ethylene carbonate^20^,^38^ in the electrolyte, or coating a protective hollow carbon sphere layer^39^ on lithium metal, to allow stable Li deposition and prevent dendrite formation could also potentially work in Na-metal battery systems.

We herein report a stable room-temperature rechargeable Na–S battery (Fig. 1a) that overcomes all of the aforementioned challenges. The battery utilizes a Na metal anode, a metal-organic framework (MOF)-derived^27^ microporous carbon polyhedron-sulfur composite (MCPS) cathode, and a liquid electrolyte comprised of a 1:1 mixture of ethylene carbonate (EC) and propylene carbonate (PC) containing 1 M NaClO_4_ salt and 1-methyl-3-propylimidazolium-chlorate ionic liquid tethered silica nanoparticle (SiO_2_–IL–ClO_4_) additives as an agent for stabilizing electrodeposition. Na–S cells with this design are shown to achieve excellent cycling performance with nearly 100 % Coulombic efficiency at higher current density and with relatively high sulfur loadings in the cathode. Reversible storage capacities of over 860 mAh g^−1^ at 0.1 C (1 C=1,675 mA g^−1^) and 600 mAh g^−1^ at 0.5 C based on active sulfur mass are reported. Even at the higher current density (0.5 C) the batteries are able to cycle stably for over 100 cycles with 0.31% capacity decay per cycle. The fundamental origins of the superior performance of the constructed Na–S cells are studied using spectroscopic tools and analysis to understand the electrochemistry at the cathode on sodiation and desodiation processes. Notably, we find that no soluble NaPS species are formed and that the diffusivity of Na^+^ into the composite cathode is consistent with expectations for solid-state transport. Altogether, these results indicate that the Na–S cells follow a different electrochemical reaction mechanism compared to traditional metal–sulfur batteries, which likely contributes to the stability and high capacity retention on cycling.

## Results

### Electrolyte stability

**Figure 1:**
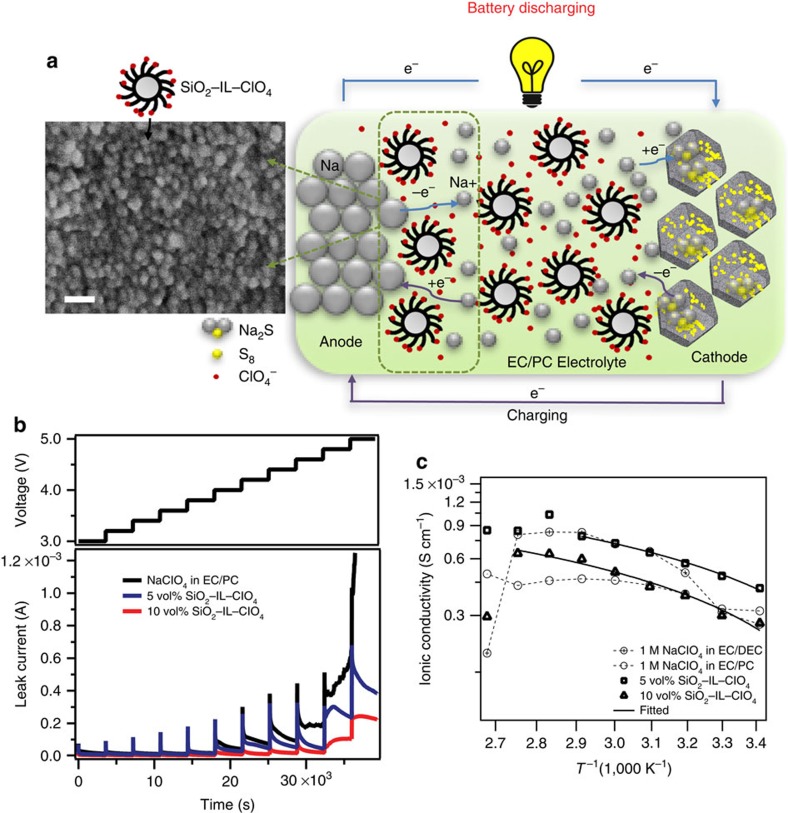
A stable sodium–sulfur (Na–S) cell. (**a**) Schematic drawing of the Na–S cell during galvanostatic cycling, using 1-methyl-3-propylimidazolium-chlorate ionic liquid tethered silica nanoparticle (SiO_2_–IL–ClO_4_) as additive in 1 M NaClO_4_ in a mixture of ethylene carbonate and propylene carbonate (EC/PC) (v:v=1:1). On the anode side, sodium atom loses electron to form sodium ion during discharge. Sodium ion diffuses inside the microporous carbon–sulfur composite and reacts with sulfur to form sodium sulfide (Na_2_S) on the cathode side, and the reverse reaction takes place during charging, where SiO_2_–IL–ClO_4_ helps stabilize sodium anode. The SEM image of the sodium metal surface cycled in a cell with 10 vol% of SiO_2_–IL–ClO_4_ in the electrolyte show for the first time that the particles form a conformal layer on the anode surface. Scale bar, 30 nm. (**b**) Constant voltage–charge profile of the Na–S cells with different volume fraction of SiO_2_–IL–ClO_4_ in the electrolyte mentioned in **a** maintained at 3.0, 3.2, 3.4…5.0 V for 1 h at room temperature. (**c**) Ionic conductivity of the Na–S cells with different volume fraction of SiO_2_–IL–ClO_4_ in the electrolyte as a function of temperature. EC/DEC represents a mixture of ethylene carbonate and diethyl carbonate (v:v=1:1). The solid lines are linear Vogel–Fulcher–Tammann (VFT) fits of the temperature dependent ionic conductivity.

The SiO_2_–IL–ClO_4_ particle additives in the electrolyte play a significant role in ensuring stable cell performance during the recharge cycle. To understand the role played by the particle additive, an electrochemical floating test was performed in the potential range from 3.0 to 5.0 V. As shown in Fig. 1b, electrolytes without particles exhibit an increase of current as the potential increases and display an unstable time-dependent current response when the potential reaches 4 V. In contrast, electrolytes containing SiO_2_–IL–ClO_4_ particle additives exhibit much lower leakage current and are stable at least up to 4.5 V. We believe that these effects stem from the same source—immobilization of a fraction of anions near the anode as a supporting electrolyte during cell recharge—as reported in lithium electrodeposition studies, where similar SiO_2_–IL–TFSI (bis(trifluoromethane)sulfonimide) particles were shown to provide orders of magnitude enhancements in the stability of lithium deposition in a PC-1M LiTFSI electrolyte^31^,^33^. Figure 1c reports the ionic conductivity as a function of temperature for EC/PC 1 M NaClO_4_ electrolytes containing different concentrations of SiO_2_–IL–ClO_4_ particles. The measurements were performed using coin cells with a Na metal electrode. It can be seen that the particles stabilize the ionic conductivities of the electrolytes, particularly at intermediate temperatures. Cells with 10 vol% particles in the electrolyte exhibit stable bulk ion transport until just below the melting point of Na metal (97.72 °C). In contrast, control cells with no particles present in the electrolyte exhibit irregular changes in conductivity with temperature, suggesting less stability. A scanning electron micrograph is provided in Fig. 1a of the sodium metal surface harvested from a cell in which 10 nm SiO_2_–IL–ClO_4_ particles were present in the electrolyte. It is obvious from the figure that the particles form a dense monolayer on the Na-metal surface, which we hypothesize is the fundamental source of the enhanced electrochemical and thermal stability. On the basis of these observations, we propose that SiO_2_–IL–ClO_4_ additive could play two roles in stabilizing the cell: (i) the tethered ionic liquid forms a mechanically robust and chemically stable SEI layer on the surface of sodium metal, which limits contact and parasitic thermal and electrochemical side reactions with the electrolyte^33^; (ii) the silica particles serve as anchor points for ClO_4_^−^ anions, which function as supporting electrolyte and reduce the electric field through the tethered anion effect discussed previously^35^,^36^ (Supplementary Fig. 1).

### Cathode characteristics

In metal–sulfur batteries, cathode design is now understood to play an important role in improving cycling performance. Due to the highly reactive nature of sodium, reaction between sodium and dissolved NaPS species is anticipated to be more vigorous, compared to lithium and LiPS. Cathode configurations that allow stronger anchoring of PS species are therefore required for Na–S cells^19^,^40^. Microporous carbon materials are thought to provide the strongest physical confinement/immobilization for sulfur and its reduction products due to their extremely small pore sizes, high surface area, and good affinity of carbon for sulfur. This material has been successfully applied to create Na–S cathodes with low sulfur loadings (32 %)^19^. Substantial increases in the sulfur loading in microporous carbon are needed to create Na–S cells that live up to the potential of this chemistry outlined in the introduction.

**Figure 2:**
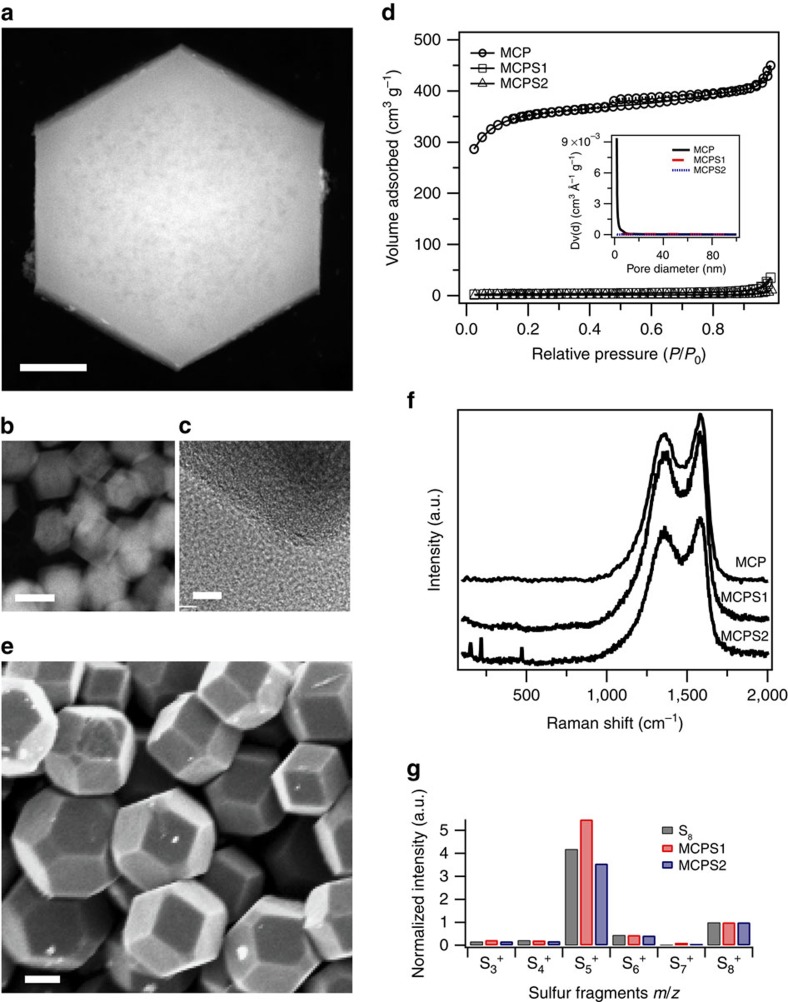
Physical characterization of the microporous carbon polyhedrons (MCP) and microporous carbon polyhedron-sulfur composites. (**a**,**b**) Scanning transmission electron microscopy (STEM) and (**c**) transmission electron microscopy (TEM) images of MCP. The TEM image shows the edge of the MCP, indicating uniform porous structure of the MCP. Scale bar, (**a**) 200 nm; (**b**) 1 μm; (**c**) 5 nm. (**d**) N_2_ adsorption–desorption isotherm and the corresponding Barrett–Joyner–Halenda (BJH) pore size distribution (inset) of MCP and MCPS composites. (**e**) Scanning electron microscopy (SEM) image of MCPS1. Scale bar, 200 nm. (**f**) Raman spectra of the MCP and MCPS composites. Three peaks in MCPS2 between 100 and 500 cm^−1^ are the signature peaks of crystalline sulfur. (**g**) Normalized positive sulfur fragmentation intensities with respect to S_8_^+^ (intensity is 1) for elemental sulfur, MCPS1 and MCPS2. m/z for S_3_^+^, S_4_^+^, S_5_^+^, S_6_^+^, S_7_^+^, S_8_^+^ are 95.916, 127.888, 159,860, 191.832, 223.804 and 255.776, respectively.

To achieve this goal, designs of the porous carbon host with homogeneous pore size distribution, high pore volume and increased surface area^41^ are needed. We employed a facile synthesis route to create well-patterned microporous carbon polyhedrons (MCP) using zeolite-type MOF (ZIF-8) rhombic dodecahedra as both the template and precursor^27^,^42^. Figure 2a–c reports scanning transmission electron microscopy and transmission electron microscopy images of the MCP, indicating a uniform microporous sponge-like texture. The abundant micropores give rise to a high Brunauer–Emmett–Teller surface area of 833 m^2^ g^−1^ calculated from the N_2_ adsorption–desorption isotherm (Fig. 2d; Supplementary Table 1), which also give rise to a high (708.5 m^2^ g^−1^) micropore surface area and pore size distribution ranging from 0.6 to 1.8 nm. To create cathodes, different amounts of sulfur were infused into the MCP and the resultant composites denoted as MCPS1 and MCPS2 with 47% and 65% sulfur loading, respectively (verified by TGA curve in Supplementary Fig. 2). The weight loss for MCPS1 due to the evaporation of sulfur occurs in a wide temperature range up to 450 °C, indicating strong nonpolar interaction between sulfur and the carbon matrix, while MCPS2 shows a two-step weight loss, representing sulfur species outside and inside the MCP, respectively. A scanning electron microscopy (SEM) image (Fig. 2e) of the as-synthesized MCPS1 suggests that the rhombic dodecahedra morphology from ZIF-8 (Supplementary Fig. 3) is well maintained after the carbonization and sulfur infusion processes, and most of the sulfur is trapped inside the micropores.

The state of sulfur inside micropores can play a significant role in the electrochemical stability of the cathode, but remains unclear^43^. Raman spectra (Fig. 2f) of MCP and MCPS composites indicate carbonization of ZIF-8 and good dispersion of sulfur in the micropores in MCPS1 as no crystalline sulfur peaks can be observed. Sulfur fragments obtained from different sulfur-containing species were evaluated by direct analysis in real-time mass spectra (DART-MS). Normalized intensities for positively charged sulfur fragments are summarized in Fig. 2g. S_8_^+^ was identified both from elemental sulfur and MCPS composites, and S_5_^+^ was the dominant positively charged sulfur species in all samples. When a negative ion source was applied in DART-MS to the MCPS composites, S_3_^−^ is found to be the dominant sulfur species (Supplementary Fig. 4). Combining the dominant sulfur fragments both from positive- and negative-ion-source measurements, yields S_8_ as the main species in MCPS. This observation indicates that sulfur inside microporous carbon used in the present work is not the smaller sulfur S_2–4_ species seen by Xin *et al*.^26^ in their studies, but rather still exists as S_8_. We also note that even the results in ref. 26 may be doubtful for while Xin *et al*. used DFT simulation of different sulfur allotropes to argue that S_8_ cannot fit inside microporous carbon, considering the ring diameter, 0.69 nm, and crown-like ring structure^44^, S_8_ is actually able to be accommodated inside extremely small micropore since the pore size distribution is an average value and the shape of the micropores in carbon is typically slit-like^24^,^28^. Galvanostatic discharge experiments by Moon *et al*.^45^ show that introduction of β-monoclinic S_8_ inside a vertically aligned carbon nanotube with a diameter of 3 nm eliminates the upper (2.4 V) discharge plateau associated with formation of soluble polysulfide species in Li–S cells. This suggests that S_8_ can probably show different electrochemistry when under confinement in porous carbon.

### Electrochemical properties

**Figure 3:**
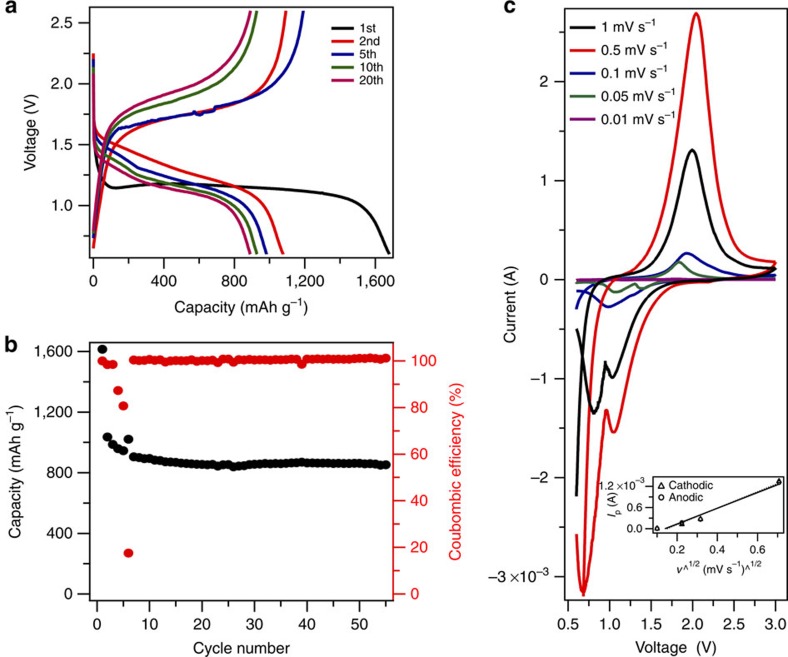
Electrochemical characterization of the Na–S cell in a carbonate electrolyte. (**a**) Electrochemical discharge and charge curves of the cell at various cycles. The tests were performed at 0.1 C for both charge and discharge in the potential range of 0.6–2.6 V versus Na/Na^+^. (**b**) Capacity and Coulombic efficiencies versus cycle number for the cell. (**c**) Cyclic voltammograms (CV) of the Na–S cells at various scan rates; inset: relation between peak cathodic and anodic currents verse square root of scan rate derived from CV.

Galvanostatic cycling experiments were performed to assess the electrochemical properties of Na–S cells in which MCPS1 is used as cathode. Results reported in Fig. 3a show that the cell exhibits a high initial discharge capacity of 1614, mAh g^−1^ at a current density of 0.1 C (1 C=1,675 mAh g^−1^). The dimple and the lower-voltage plateau at the beginning of discharge, compared with the following cycles, indicates that Na^+^ ions need to go through a barrier, which is probably related to desolvation or solvation shell distortion^46^,^47^ to accommodate the extremely small pore size to diffuse inside the micropores. The higher irreversible capacity is partially attributed to initial SEI formation and electrolyte decomposition. The reversible discharge plateau in the following cycles ranges from 1.6 to 1 V. The lower voltage is consistent with direct formation of Na_2_S/Na_2_S_2_, without creation of intermediate soluble NaPS species. A reversible discharge capacity of 800 mAh g^−1^ is stably achieved for 50 cycles (Fig. 3b).

In contrast, when TEGDME, which has high solubility for NaPS, is used as the electrolyte solvent, shuttling is observed in the voltage profile (see Supplementary Fig. 5) and corrosion of the sodium anode is readily seen from post-mortem studies (Supplementary Fig. 6). This has been known for some time to be the cause of cell failure in sodium–sulfur batteries using ether-based electrolytes^14^,^18^,^48^,^49^,^50^,^51^,^52^,^53^. Those batteries can neither bear high current densities (1/64C is employed in ref. 48)^48^,^49^ nor exhibit satisfactory cycle life^50^,^51^,^52^,^53^. Even though NaNO_3_ is used as an electrolyte additive to passivate sodium metal—with the expectation that it is as effective as LiNO_3_ on passivating lithium anode in Li–S batteries^54^—the cells do not achieve outstanding performance in any of the studied configurations. A similar result is found when MCPS2 is used as cathode with the EC/PC based electrolyte, where data reported in Supplementary Fig. 7 indicate that higher-order NaPSs is formed and react with the carbonate electrolyte. Thus, carbonate-based electrolytes are shown to be incompatible with metal–sulfur batteries if insufficient care is taken to sequester NaPS in the cathode^43^,^55^. These results therefore clearly show that the electrochemistry of sulfur in micporours carbon is affected by subtle features related the solubility of PS in electrolyte; competition between the affinity of sulfur for the microporous carbon and electrolyte determines whether and what intermediate sulfide specie is observed.

Low self-discharge is another required feature of a stable electrochemical energy-storage device^56^,^57^. Metal–sulfur batteries, unfortunately, have strong self-discharge behaviour in ether-based electrolytes due to the formation and dissolution of metal polysulfides. Comparison of the initial discharge voltage profile (Supplementary Fig. 5c,d) between fully charged Na–S cells based on carbonate electrolytes and the MCPS1 composite cathodes show little difference after 10-min and 2-week resting times, indicating relatively small decrease in capacity (<22%). Na–S cells containing TEGDME electrolyte and MCPS1 composite cathodes exhibit a 42% capacity decay after 2 weeks. In contrast Na–S cells based on a physical sulfur–carbon blend cathode exhibit an immediate voltage drop and short circuit. This observation confirms the importance of confinement of sulfur in MCP for minimizing self-discharge and parasitic internal chemical reactions in the cell.

In the carbonate electrolyte, interaction between sulfur and the microporous carbon appears to be strong enough to completely prevent sulfur loss to the electrolyte. This raises the possibility that sulfur undergoes a solid-state electrochemical reaction in the microporous carbon. To investigate this possibility, cyclic voltammetry measurements were performed at various scan rates. Results reported in Fig. 3c clearly shows that a two-electron transfer process occurs in the discharge cycle. The reduction peaks are seen to shift towards more negative values and oxidation peak towards more positive values with increasing scan rate, indicative of an electrochemical process in which mixed kinetics of charge transfer process and diffusion of electroactive species control^58^. The cathodic and anodic peak currents are also seen to be similar in magnitude, which indicates good reversibility and similar reaction mechanisms occurring during charge and discharge. Finally, the peak current is found to increase linearly with the square root of scan rate (Fig. 3c), which is a classical characteristic of a diffusion-limited process^59^.

**Figure 4:**
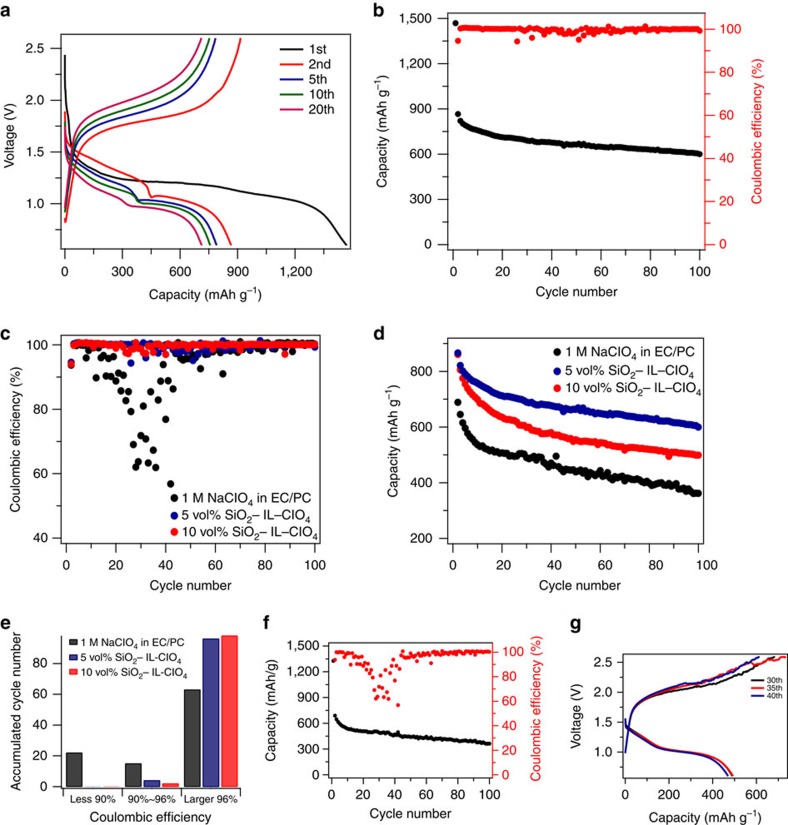
Galvanostatic cycling performance of the Na–S cell in a carbonate electrolyte with different amounts of SiO_2_–IL–ClO_4_. (**a**) Electrochemical discharge and charge curves of the cell at various cycles with 5 vol% of SiO_2_–IL–ClO_4_ in the electrolyte. The tests were performed at 0.1 C for the first discharge and 0.5 C for the following cycles in the potential range of 0.6–2.6 V versus Na/Na^+^. (**b**) Capacity and Coulombic efficiencies versus cycle number for the cell with 5 vol% of SiO_2_–IL–ClO_4_ in the electrolyte. (**c**) Coulombic efficiency and (**d**) capacity versus cycle number for the cell with different amounts of SiO_2_–IL–ClO_4_ in the electrolytes, respectively, at a current density of 0.5 C. (**e**) Electrolyte stability analysis for the three cases in **c** in term of Coulombic efficiency for the first 100 cycles. (**f**) cycling performance and (**g**) voltage profile of the cell without SiO_2_–IL–ClO_4_ in the electrolyte at 0.5 C.

A superficial assessment of the cycling results in Fig. 3a would conclude that the Na–S cells cycle well. More careful scrutiny of the large drop in Columbic efficiency seen in Fig. 3b at around the 6th cycle and the slight ripple in the charge profile at the 10th cycle shown in Fig. 3a reveal serious stability problems with the Na–S cells (see Supplementary Fig. 8). Measurements at higher current density show that the cells become progressively more unstable and the effects seen in Fig. 3a,b become more severe. Because the unstable operation is only evident during cell recharge, we suspected that it originated from unstable Na deposition and/or side reactions of the freshly created Na surface area with the electrolyte^60^,^61^. Motivated by the earlier work by Lu *et al*.^31^,^32^,^33^, which showed that SiO_2_–IL–TFSI particles can stabilize electrodeposition of Li by both the tethered anion mechanism and by protecting Li metal against parasitic side reactions with liquid electrolytes, we investigated the effect of SiO_2_–IL–ClO_4_ nanoparticles as electrolyte additives for Na–S batteries. Previous studies have reported that at 10% SiO_2_–IL–TFSI particle additives in 1 M NaTFSI in PC electrolytes stabilized the charging process in Na-CO_2_/O_2_ cells^62^; leading to rechargeable batteries based on even that novel chemistry. In the present studies, either 5 vol% or 10 vol% of the SiO_2_–IL–ClO_4_ was added to the electrolyte as a stabilizer and the cell response in galvanostatic cycling experiments compared with those obtained from control experiments in which the IL-tethered particles were not present. Consistent with the previously reported results, it is seen that as little as 5 vol% of the SiO_2_–IL–ClO_4_ additive could stabilize charging to a large extent. Figure 4a,b report the voltage profile and cycling stability of these cells. The first discharge is performed at 0.1 C to fully activate the electrode. A discharge capacity of around 866 mAh g^−1^ is achieved initially and maintained to 600 mAh g^−1^ at the 100th cycle, indicating a small capacity decay of 0.31% per cycle, which is comparable to current Li–S batteries at the same C rate. The Coulombic efficiencies for the batteries with and without SiO_2_–IL–ClO_4_ are compared in Fig. 4c–g to evaluate their stability. It is apparent that cells without SiO_2_–IL–ClO_4_ show diverging Coulombic efficiency between the 10th to 60th cycle, while cells with only small amounts of SiO_2_–IL–ClO_4_ exhibit improved Coulombic efficiency to over 90% each cycle, which is enhanced with increasing SiO_2_–IL–ClO_4_ amount. A benefit of the improved charging stability of the cells is that their cycling performance is enhanced over multiple discharge cycles as shown in Fig. 4d. We tentatively attribute this effect to a reduction in electrolyte loss as a result of side reactions with the anode during cell recharge.

### Reaction mechanism

**Figure 5:**
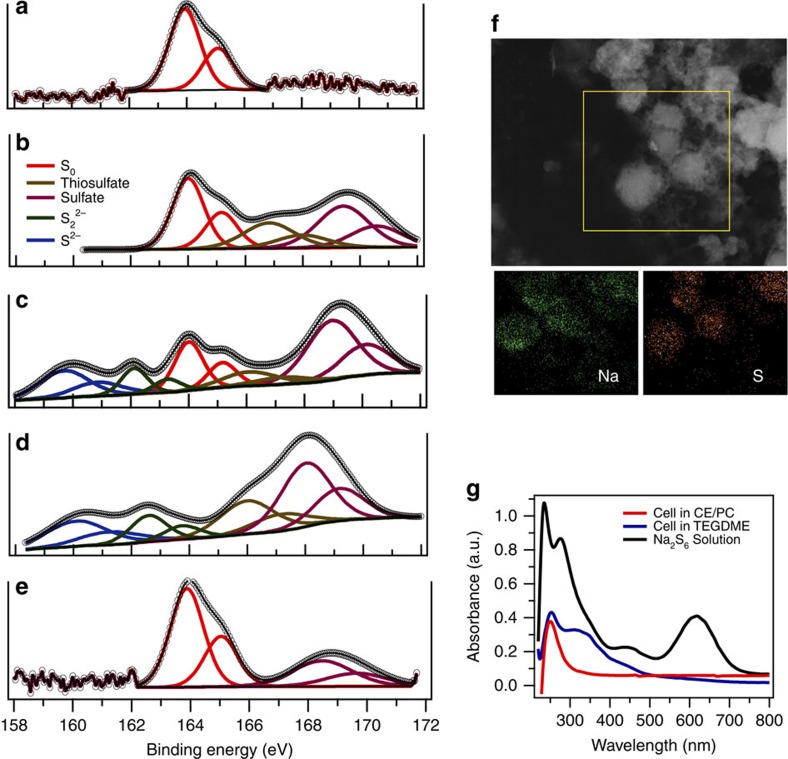
Post-mortem characterization of the MCPS1 cathodes in carbonate electrolytes. (**a**–**e**) *Ex situ* XPS spectra of S 2p in MCPS1 cathodes at pristine and different cycling states in a carbonate electrolyte. (**a**), pristine cathode. Cell was discharged to (**b**) 1.4 V, (**c**) 1 V and (**d**) 0.6 V and was recharged to (**e**) 2.6 V at the first cycle, respectively. Cathodes were disassembled in an argon-filled grove-box and washed with electrolyte solvent before characterization. (**f**) STEM image and EDX maps of the MCPS1 cathode after first discharge. The cathode was washed with electrolyte solvent and sonicated to form a homogeneous suspension in a sealed vial. EDX, energy dispersive X-ray. (**g**) Ultraviolet–vis spectra of the cathodes solutions cycled in different electrolytes after 10 cycles at 0.1 C. The MCPS1 cathodes cycled in different electrolytes were soaked in 2 ml TEGDME for 4 days to extract PS species. For the Na_2_S_6_ solution, 1 M Na_2_S_6_, which is synthesized by mixing Na_2_S and sulfur in a stoichiometric ratio of 2:6 in TEGDME. It was diluted 200 times and subjected to test. Peak assignment: S^2−^ and S_2_^2−^, ∼260 nm; S_6_^2−^, 340 nm and 450 nm; S_3_^2−^, 330 nm; S_3_.^−^: 610 nm (ref. 65).

To investigate the discharge reaction mechanism, X-ray photoelectron spectroscopy (XPS) was applied on the cathode side after galvanostatic cycling at different stages of discharge/charge to study the species formed at each stage (Fig. 5a–e). The pristine cathode exhibits an elemental-state sulfur doublet with S2p 2/3 at 164 eV. When the cell is discharged below 1 V, S2p 2/3 peaks at 162.1 and 160 eV representing Na_2_S_2_ and Na_2_S rise. The peak beyond 166 eV is probably due to thiosulfate/sulfate complex species originating from oxidized sulfide species^63^. On full discharge, the elemental sulfur peak disappeared; only the sulfide peaks remain, suggesting the final discharge product is Na_2_S. This can explain why a higher capacity is achieved compared with high-temperature Na–S batteries, where the final discharge product is Na_2_S_*x*_ (*x*≥3) because of the phase limitation^16^. Energy dispersive X-ray analysis of the cathode after full discharge reveal the atomic ratio of Na and S is about 2.1, consistent with an almost full reduction from S to Na_2_S, and sulfur intensities concentrated inside the micropores and suggesting the solid-state reaction (Fig. 5f; Supplementary Fig. 9). To further understand the reaction mechanism, an organic conversation technique was utilized to characterize the reaction species during initial cycling in both EC/PC and TEGDME electrolytes. In this approach, highly reactive sulfide species are first converted to their stable analogue benzyl sulfides (BzS_*x*_, *x*=1–5)^43^,^64^, and nuclear magnetic resonance (NMR) spectroscopy of the analogues applied to analyse the organic molecules (Supplementary Fig. 10 and Supplementary Note 1). Remarkably, chemical shifts representing Bz_2_S were observed in the whole process for the cell utilizing EC/PC carbonate electrolyte and in the first discharge process for the cell based on the TEGDME electrolyte. In contrast, high-order BzS_*x*_ are clearly observed in recharged cathodes in TEGDME. Ultraviolet–visible (ultraviolet–vis) spectra of a dilute Na_2_S_6_ solution, battery cathodes after 10 cycles in TEGDME, and carbonate electrolyte soaked in TEGDME are shown in Fig. 5g. In the carbonate electrolyte, there are only insoluble S_2_^2−^ or S^2−^ formed, while the high-order soluble PS are formed when the cells are cycled in TEGDME^65^. This again confirms our hypothesis that the MCPS cathode coupled with carbonate electrolyte undergoes solid-state reaction with no soluble intermediate polysulfide formed in Na–S batteries.

**Figure 6:**
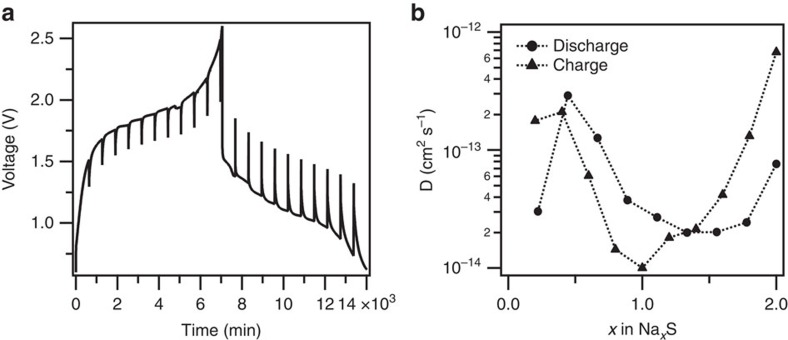
Diffusivity analysis of the Na–S cell. (**a**) Galvanostatic intermittent titration technique (GITT) curves of MCPS1 in EC/PC electrolyte and (**b**) Diffusion coefficients derived from **a**.

On the basis of the spectroscopic study above, one may more firmly hypothesize that the electrochemistry in the cathode reaction occurs completely inside the MCPS, which means that both the transport of Na^+^ into the cathode and the electrochemical reaction with sulfur in the cathode progress as solid-state processes. To verify this hypothesis, we first extract an approximate value for the Na^+^ diffusivity in the cathode based on the electrochemical data. Measurements utilizing a galvanostatic intermittent titration technique (GITT) was performed by discharging the cell for 30 min at 0.1 C followed by a 10-h relaxation (Fig. 6a). The diffusion coefficient (Fig. 6b) at different stages during reversible charging and discharging can be calculated from the transient voltage response using an expression developed by Weppner and Huggins^66^ for solid-state diffusion processes in batteries. The Na^+^ diffusivity deduced from this analysis is found to be the lowest in the region where the discharge profile exhibits a clear plateau, consistent with the idea that a kinetics-controlled mechanism is overlayed on the discharge. In addition, the equilibrium potential determined at the end of each titration step changes very slightly and all below 2 V, suggesting the formation of Na_2_S_*x*_ (*x*≤2). The reason why the solid-state reaction occurs only in microporous carbons with small pore sizes, but not in other carbon materials is open to argument and will likely be a subject for many future studies. On the basis of the empirical evidence in the present study, we attribute the difference to the stronger interactions between sulfur/sulfur species in the carbon relative to the strength of sulfur/sulfur species solvation due to their poor solubility in carbonate electrolytes^67^ and to the short electronic transport lengths, which permit good active material utilization during normal battery operation even under slow solid-state transport kinetics.

## Discussion

We report an example of a room-temperature, rechargeable Na–S battery that can be cycled stably with high Coulombic efficiency at low and moderate current densities. The battery utilizes a micoporous carbon/sulfur composite in the cathode and an EC/PC-1M NaClO_4_ electrolyte. The combination of cathode substrate and electrolyte are shown to provide sufficiently strong association of sulfur in the cathode to confine the electrochemical reactions in the cathode to an all-solid-state process in which Na_2_S appears to be the only product. We use both spectroscopic and analytical tools to show that for the carbons used in the present work, sulfur remains as S_8_ and that the cathode reaction occurs inside the microporous carbon composite. An additional problem associated with instability during the recharge process of Na–S cells operated at moderate and high current density was identified and resolved using SiO_2_–IL–ClO_4_ particles as additives in the electrolytes. Electron microscopy and electrochemical analysis indicate that the particles form a dense protective coating on the Na anode and stabilize deposition of sodium by at least two mechanisms. First, they form a particle-rich, mechanically strong SEI layer that protects sodium metal from parasitic side reactions with the liquid carbonate electrolyte. Second, they appear to utilize a previously reported tethered anion effect to stabilize deposition of Na. Our finding underscores the benefits of micorporous carbon–sulfur composite and nanoparticles for guiding new material designs for inexpensive rechargeable metal–sulfur batteries. Further investigations are needed to fully understand the interaction of microporous carbon and sulfur species as well as the specific role SiO_2_–IL–ClO_4_ plays on metal anode protection.

## Methods

### Materials synthesis

MCPS and SiO_2_–IL–ClO_4_ electrolyte were synthesized according to the previous methods with modifications^27^,^32^. Briefly, the synthesis of MCPS is the same except the final sulfur infusion step. A sealed Pyrex tube was used to hold samples and a ramp rate of 1 °C min^−1^ was used for both heating and cooling. The final mass fraction of sulfur in the composites was determined by TGA (Q5000 IR Thermogravimetric Analyzer). The synthesis of SiO_2_–IL–ClO_4_ was the same as well except the anion exchange step. In this work, NaClO_4_ was used to as anion exchange source.

### Material characterization

The morphology and elemental mappings of the materials were studied using a FEI Tecnai F20 Transmission Electron Microscope and A LEO 1550 high-resolution SEM. The nitrogen adsorption–desorption isotherms of the MCP and MCPS were obtained with a Brunauer–Emmett–Teller (Micromeritics ASAP2020). AccuTOF DART was used to get mass spectra for sulfur and MCPS composites. Raman spectra were collected using a Renishaw InVia Confocal Raman Microscope (*λ*=488 nm). ^1^H NMR spectra were taken by Inova-400 Spectrometer. Ultraviolet–vis spectra were collected by Shimadzu UV–Vis–NIR Spectrometer. XPS measurements were performed with a Surface Science SSX-100 spectrometer using a monochromatic Al Kα source (1486.6 eV). Non-linear least squares curve fitting was applied to high-resolution spectra, using CasaXPS software.

### Electrochemical measurements

The cathodes were prepared with MCPS1 or MCPS2, carbon black (Super-P, TIMCAL), and polymer binder (poly(vinylidene difluoride), PVDF, Aldrich) in a weight ratio of 8:1:1. A carbon-coated aluminium foil (0.018 mm in thick, 1.27 cm in diameter, MTI Corp.) was used as the current collector. The typical thickness of the active material film is ∼20 μm and sulfur loading is around 0.73 to 1 mg. Sodium foil (Alfa Aesar) was used as the counter and reference electrode. A glass fibre filtre paper (Watchman 934-AH) was used as separator. 80 μl 1 M sodium perchlorate (NaClO_4_) in a mixture ethylene carbonate (EC) and diethyl carbonate (DEC; v:v=1:1) or in tetraethylene glycol dimethyl ether (TEGDME) or in a mixture of EC and propylene carbonate (PC; v:v=1:1) with different amount of SiO_2_–IL–ClO_4_ were used as electrolyte for the cells. Cell assembly was carried out in an argon-filled glove-box (MBraun Labmaster) by using coin cell 2032 type. The room-temperature cycling characteristics of the cells were evaluated under galvanostatic conditions using Neware CT-3008 battery testers and electrochemical processes in the cells were studied by cyclic voltammetry using a CHI600D potentiostat. Electrochemical impedance and floating tests were conducted by using a Solartron Cell Test System model 1470E potentiostat/galvanostat. Ionic conductivities were measured using a Novocontrol N40 broadband dielectric spectrometer.

For post-mortem studies, cells were disassembled in an argon-filled glove-box and the electrodes were harvested and rinsed thoroughly with the electrolyte solvent before analysis.

### Data availability

The data that support the findings of this study are available from the corresponding authors upon request.

## Additional information

**How to cite this article:** Wei, S. *et al*. A stable room-temperature sodium–sulfur battery. *Nat. Commun.* 7:11722 doi: 10.1038/ncomms11722 (2016).

## Supplementary Material

Supplementary Information Supplementary Figures 1-10, Supplementary Table 1, Supplementary Note 1 and Supplementary References
