# Supplementary material for: A stable room-temperature sodium–sulfur battery
Source: Nat Commun. 2016 Jun 9;7:11722. doi: 10.1038/ncomms11722 (PMC4906167; doi:10.1038/ncomms11722)
Supplement: Supplementary Information — Supplementary Figures 1-10, Supplementary Table 1, Supplementary Note 1 and Supplementary References [file ncomms11722-s1.pdf]

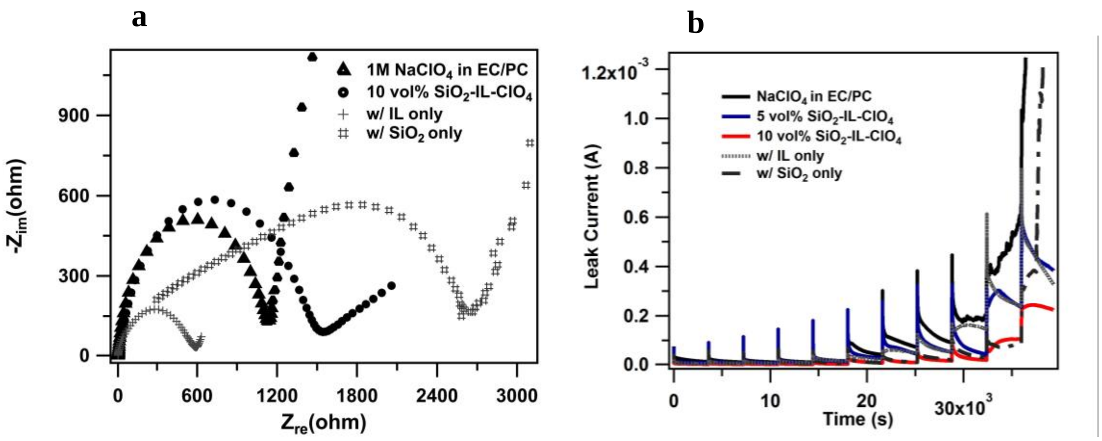

Supplementary Figure 1: Impedance spectra (a) and Floating test (b) of the Na-S cells with different electrolyte additives. The black triangles in **a** report the Nyquist plot of the Na-S cell in 80 µl 1M $NaClO_4$ in EC/PC, whereas the data represented by black circles are the corresponding to the cell in 10 vol% of SiO_2-IL-ClO_4 in the electrolyte mentioned above. The plus and number signs represent electrolytes contain IL and silica that are equivalent amount of 10 vol% of SiO_2-IL-ClO_4 in the electrolyte respectively. The case with only SiO_2 shows a large increase in interfacial resistance, indicating the formation of a physical barrier in the electrode-electrolyte interphase, while the case with only IL decreases the interfacial resistance a lot, resulting from the high conductivity of the IL. **b**, cell with IL or SiO_2 only as the electrolyte additive shows improved stability compared to the cell with no additive, but both of them are not as good as cell with SiO_2-IL-ClO_4 as the additive.

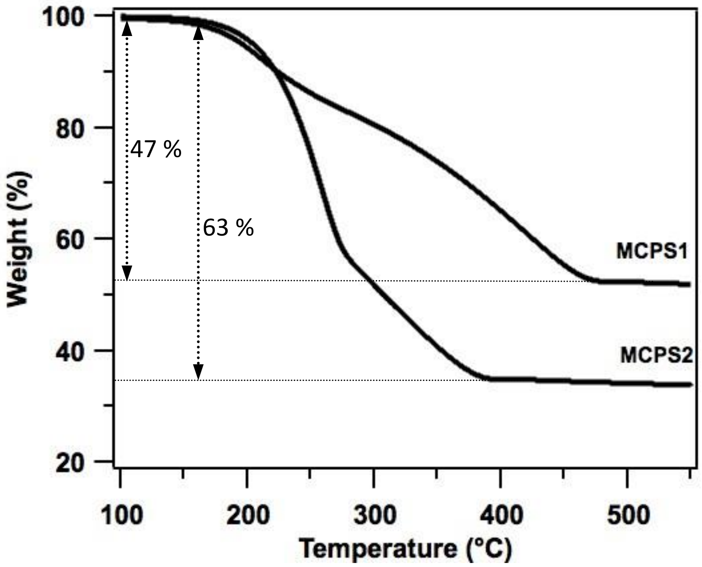

Supplementary Figure 2: Thermogravimetric analysis (TGA) of the MCPSs with different sulfur loadings under N_2 flow.

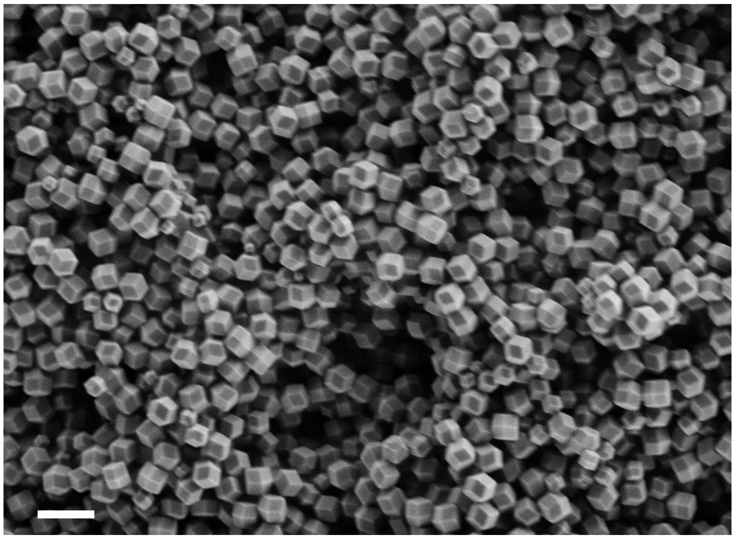

Supplementary Figure 3: Scanning electron microscopy (SEM) image of the porous zeolite-type MOF (ZIF-8) rhombic dodecahedra. These polyhedrons are about 1 μm in size with smooth surface. Scale bar, 2 μm.

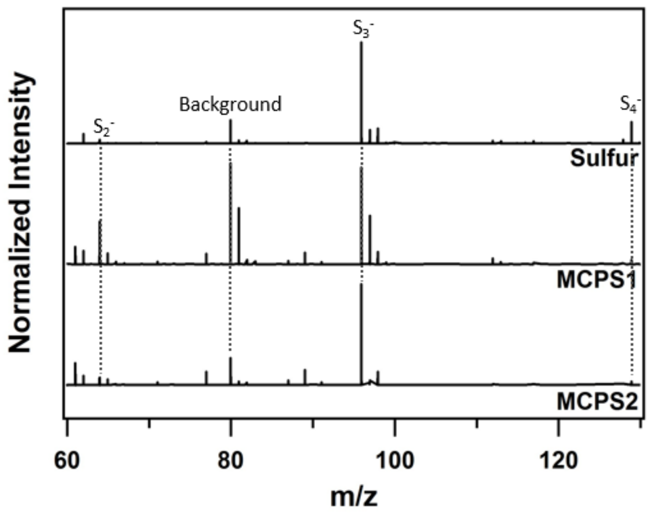

Supplementary Figure 4: Direct analysis in real time mass spectra (DART-MS) of MCP composites and sulfur by a negatively charged ion source. S_3^- is the dominant sulfur species in all cases.

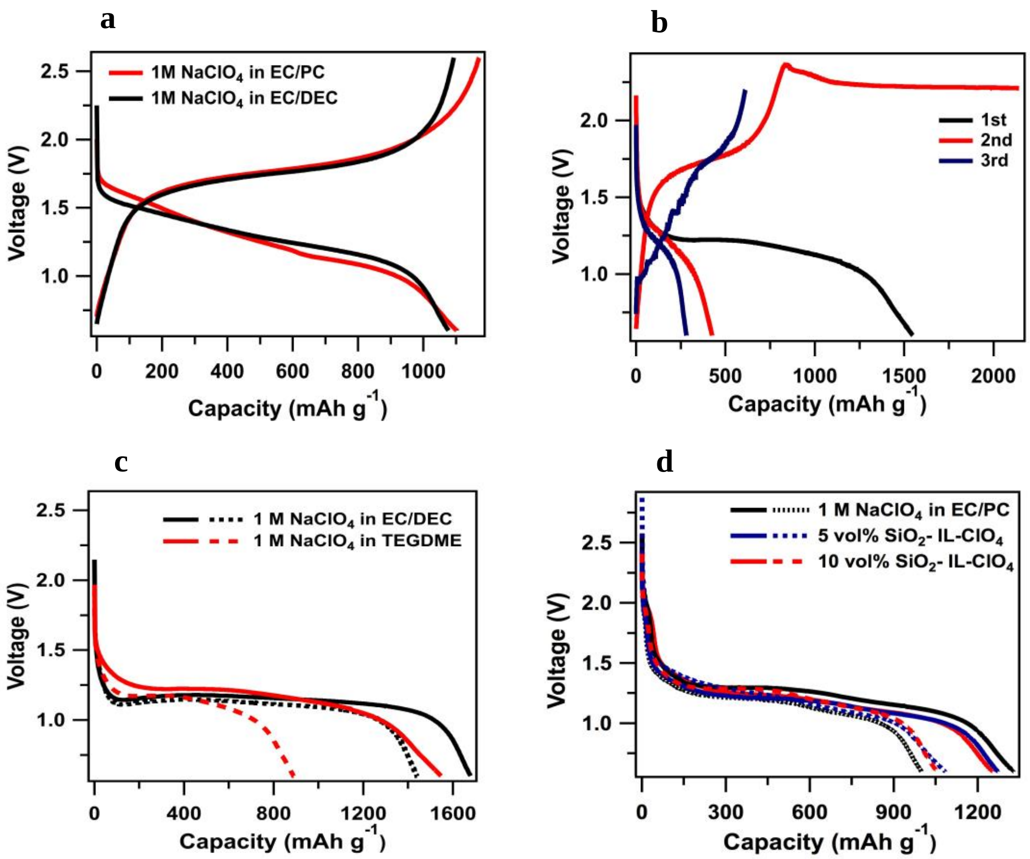

Supplementary Figure 5: Voltage profile of the Na-S cells (**a**) in different carbonate electrolytes (reversible profile, 2nd cycle); (**b**) in the TEGDME-based electrolyte; (**c**, **d**) with different resting time (solid line: rest 10 mins; dash line: rest 2 weeks) after cell assembly and before galvanostatic cycling test in the different electrolytes studied in this work. Tetraethylene glycol dimethyl ether (TEGDME) is an ether-based electrolyte used in Li-S and Na-S batteries due to its higher solubility to PS[1, 2], however, carbonate electrolytes are suggest to be non-soluble to PS[3]. When using MCPS1 as cathode in TEGDME electrolyte, although the first discharge voltage profile is comparable to that in carbonate electrolytes, server shuttling is observed during recharge process. The self-discharge rates for the cells in different type of carbonate electrolyte are within 22% and for the cells in TEGDME, it is around 42 %. The current density is 0.1C for both charge and discharge

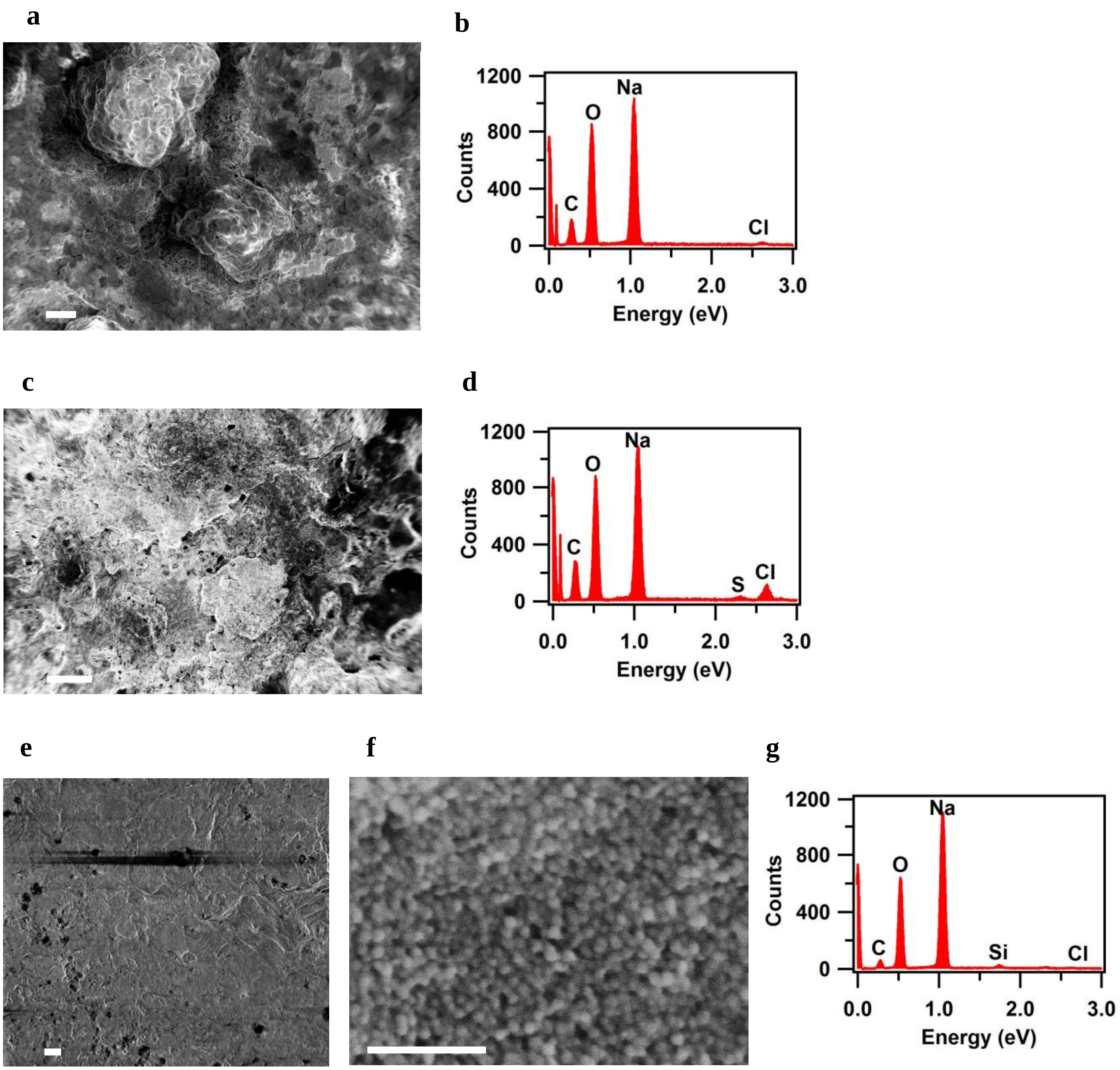

Supplementary Figure 6: Scanning electron microscopy (SEM) image and energy dispersive spectra (EDS) maps of the sodium anodes after 10 cycles. SEM image and EDS maps of the sodium anode in 80 µL 1M NaClO4 in EC/DEC electrolyte (**a**, **b**); 80 µL 1M NaClO4 in TEGDME electrolyte (**c**, **d**); in 1M NaClO4 in EC/PC electrolyte with 10 vol% SiO_2-IL-ClO_4 (**e**, **f**, **g**). The cells were dissembled after 10 cycles of charge and discharge at 0.1C in an argon filled glovebox and the anodes were washed with electrolyte solvent before characterization. Sulfur signals were found in EDS spectra for the anode cycled in TEGDME, indicating that polysulfides are formed in electrolyte and attack sodium anode. Scale bar, **a**, 100 µm; **c**, 100 µm; **e**, 2 µm; **f**, 100nm.

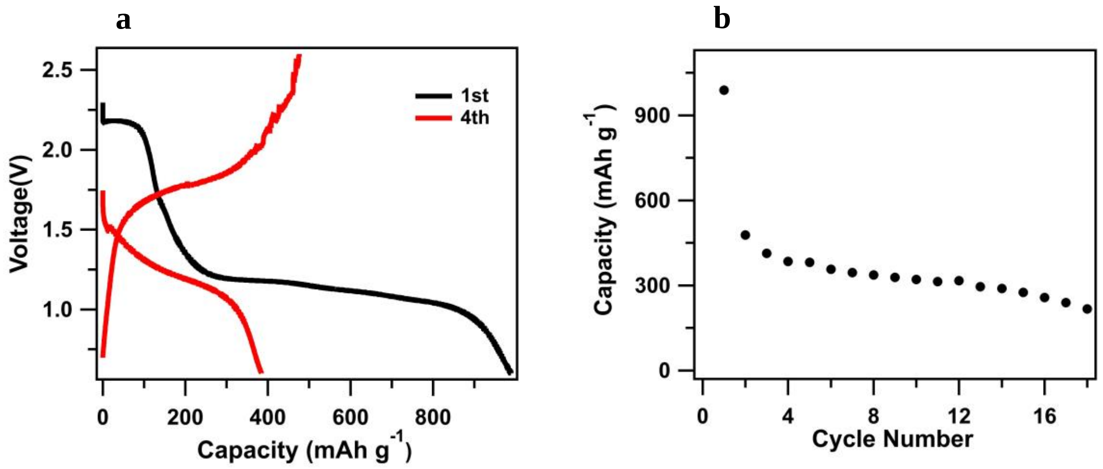

Supplementary Figure 7: Electrochemical characterization of the MCPS2 in a carbonate electrolyte. (**a**) Electrochemical discharge and charge curves of the cell at 0.1C. The two discharge plateau in the first cycles indicates sulfur outside and inside the MCPS2. The disappearance of the higher discharge voltage plateau suggests that the elemental sulfur outside MCP reacts irreversibly with carbonate electrolyte. (**b**) Capacity verses cycle number by using MCPS2 as the cathode.

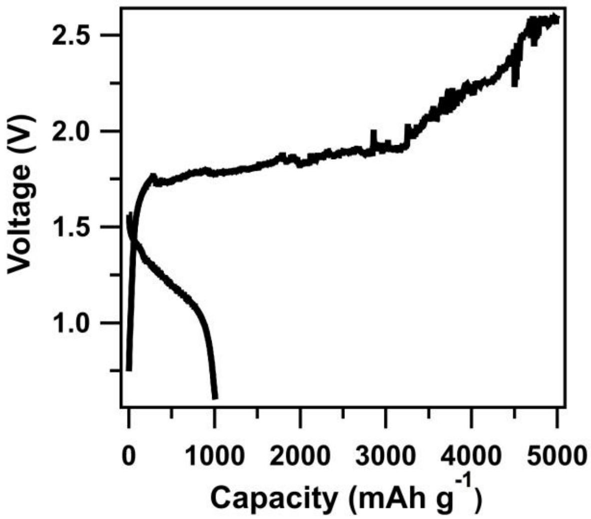

Supplementary Figure 8: The sixth cycle voltage profile of the cell in mentioned in Figure 3a and b. Severely instable charging was observed.

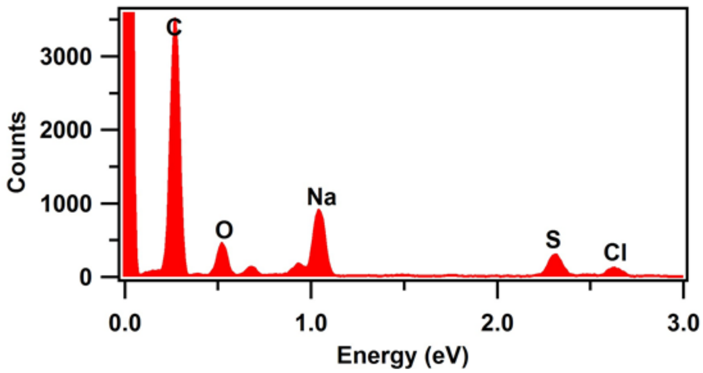

Supplementary Figure 9: EDX spectra of the MCPS1 cathode after first discharge to 0.6 V.

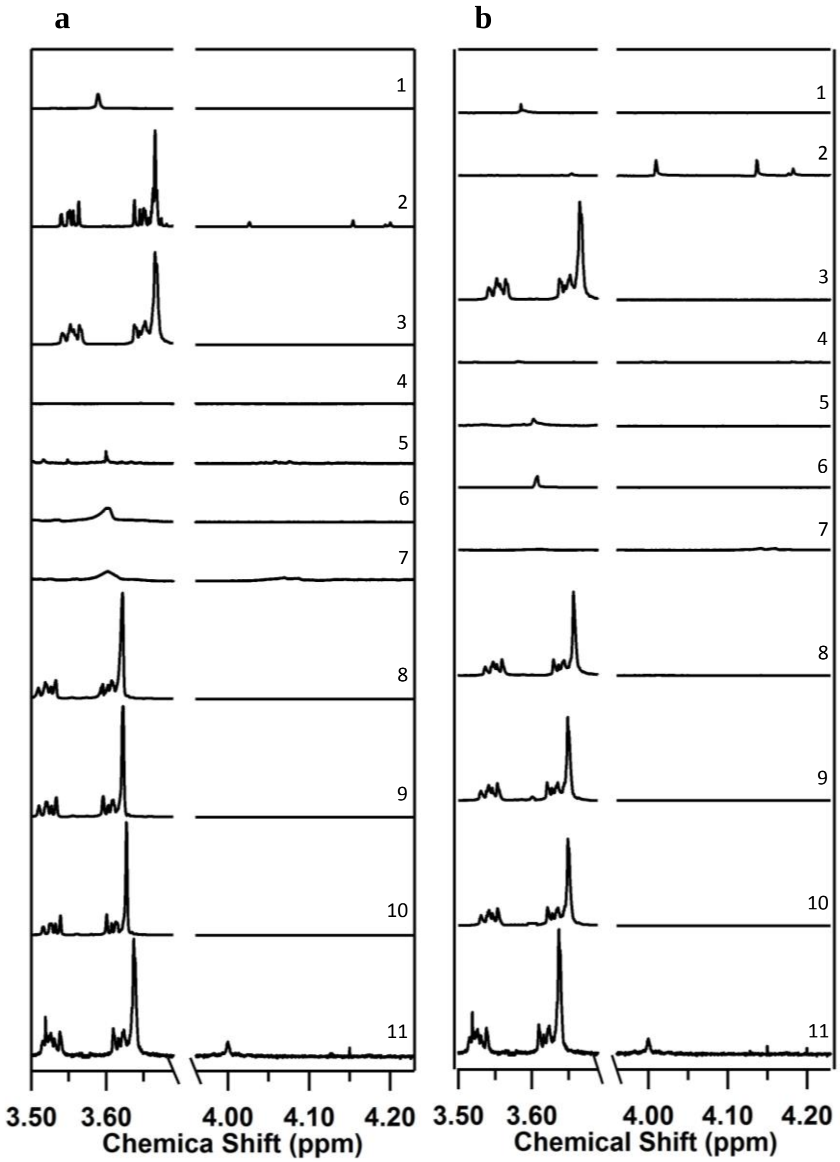

Supplementary Figure 10: ^{1}H NMR spectra of the converted benzyl sulfide species from sodium sulfide species and lithium sulfide species. (**a**) ^{1}H NMR spectra of benzyl sulfide species converted from 1: Na_2S powder; 2: Na_2S_6 solution in TEGDME; 4-6: MCPS1 cathodes discharged in the EC/DEC electrolyte to 1.4, 1, 0.6 V respectively; 7: MCPS1 cathodes at fully recharged state in EC/DEC electrolyte; 8-10: MCPS1 cathodes discharged in the TEGDME electrolyte to 1.4, 1, 0.6 V respectively; 7: MCPS1 cathodes at fully recharged state the TEGDME electrolyte. 3: ^{1}H NMR spectra of TEGDME (**b**) ^{1}H NMR spectra of benzyl sulfide species converted from 1: Li_2S powder; 2: Li_2S_3 solution 4-6: MCPS1 cathodes discharged in the EC/DEC electrolyte to 1.4, 1, 0.6 V respectively; 7: MCPS1 cathodes at fully recharged state in EC/DEC electrolyte; 8-10: MCPS1 cathodes discharged in the TEGDME electrolyte to 1.4, 1, 0.6 V respectively; 7: MCPS1 cathodes at fully recharged state the TEGDME electrolyte. 3: ^{1}H NMR spectra of TEGDME.

Samples	BET Total Surface Area (m^2 g^{-1})	Pore Volume (cm^3 g^{-1})	Micropore Surface Area (m^2 g^{-1})	DFT Pore Size Distribution (nm)	Conductivity (S cm^{-1})	I_g/I_d
MCP	833.3781	0.695505	708.4812	0.6~1.8[4]	$9.66*10^{-3}$	1.14
MCPS1	11.5262	0.054189	2.3499	n.a.	$6.13*10^{-3}$	1.14
MCPS2	3.2628	0.017352	1.1322	n.a.	$5.22*10^{-3}$	1.08

Supplementary Table 1: Physical characteristics of the MCP and MCPS. DFT: Density function theory method used to determine microspores. I_g/I_d is the ratio used to determine the amount of graphitic carbon verse disordered carbon in the composite, which are the normalized peak ratio of G band to D band in Raman Spectra.

Supplementary Note 1

In both sodium and lithium-sulfur batteries studied in this work, chemical shift of Bz_2S (3.60 ppm) was the only sulfide species observed for the cathodes cycled in the carbonate electrolyte. In TEGDEM electrolyte, although Bz_2S chemical shift was only found in the cathode at discharged stages, chemical shifts of Bz_2S_3 (4.0 ppm), Bz_2S_4 (4.15 ppm) and Bz_2S_5 (4.2 ppm) were observed at recharge stage. The phenomena indicate that sulfur (S_8) in microporous is able to reduce to solid sulfide species directly without forming soluble polysulfides. In a polysulfide soluble TEGDME electrolyte, recombination and disproportion reactions[5] occur during recharge process leading to the formation of soluble high-order polysulfides, which tentatively indicates that sulfur in microporous carbon is S_8. When using a non-solvent for polysulfide electrolyte (EC/DEC), sulfur in microporous carbon can reversibly undergo solid-state reaction to form Na_2S or Li_2S. Chemical shifts of TEGDME may change if benzyl sulfide co-exists probably dues to interaction with aromatic benzene rings[6]. Cathodes cycled at different stages were soaked in a mixture of benzyl chloride and DME and allowed to sit for four days. After that, solvents were allowed to evaporate from the samples. The samples were then mixed with chloroform-d and filtered out of impurities (mainly carbon black on the cathode). They were then transferred to NMR tube and subjected to NMR test.

Supplementary References

1. Manthiram, A., Fu, Y., Chung, S.-H., Zu, C. & Su, Y.-S. Rechargeable Lithium–Sulfur Batteries. *Chemical Reviews* **114**, 11751-11787 (2014).
2. Manthiram, A. & Yu, X. Ambient Temperature Sodium–Sulfur Batteries. *Small* **11**, 2108-2114 (2015).
3. Wei, S., Ma, L., Hendrickson, K.E., Tu, Z. & Archer, L.A. Metal–Sulfur Battery Cathodes Based on PAN–Sulfur Composites. *Journal of the American Chemical Society* **137**, 12143-12152 (2015).
4. Wu, H.B. et al. Embedding Sulfur in MOF-Derived Microporous Carbon Polyhedrons for Lithium–Sulfur Batteries. *Chemistry – A European Journal* **19**, 10804-10808 (2013).
5. Rauh, R.D., Shuker, F.S., Marston, J.M. & Brummer, S.B. Formation of lithium polysulfides in aprotic media. *Journal of Inorganic and Nuclear Chemistry* **39**, 1761-1766 (1977).
6. Cubberley, M.S. & Iverson, B.L. 1H NMR Investigation of Solvent Effects in Aromatic Stacking Interactions. *Journal of the American Chemical Society* **123**, 7560-7563 (2001).
